# Myrmecophily Under X-Rays: The Exceptional Brain of an Exceptional Beetle, *Paussus favieri* (Coleoptera, Carabidae, Paussinae)

**DOI:** 10.3390/insects17070701

**Published:** 2026-07-06

**Authors:** Francesco Sirotti, Maurizio Muzzi, Alessia Sanna, Marco Rossi, Andrea Di Giulio

**Affiliations:** 1 Department of Science, University Roma Tre, Viale G. Marconi, 00146 Rome, Italy; 2 LTTA-Electron Microscopy Center, University of Ferrara, 44121 Ferrara, Italy; 3 Department of Occupational and Environmental Medicine, Epidemiology and Hygiene, Italian Workers’ Compensation Authority (INAIL), Monte Porzio Catone, 00078 Rome, Italy; 4 CNIS, Sapienza University of Rome, Piazzale Aldo Moro 5, 00185 Rome, Italy; 5 Department of Basic and Applied Sciences for Engineering, Sapienza University of Rome, Via Antonio Scarpa 14, 00161 Rome, Italy; 6 NBFC, National Biodiversity Future Center, 90133 Palermo, Italy

**Keywords:** Ant Nest Beetles, flanged bombardier beetles, Paussini, neuroanatomy, neuropils, micro-CT, X-rays

## Abstract

Among myrmecophilous insects, beetles represent the most specialised and diverse group. Myrmecophily is a complex evolutionary strategy encompassing a wide spectrum of interactions with ants, ranging from occasional to obligate relationships, and from mutualistic associations (e.g., trophobionts) to fully parasitic symbioses (social parasites). One of the most remarkable examples of an obligate ant parasite is *Paussus favieri* Fairmaire,1851 (Carabidae, Paussinae, Paussini), a West-Mediterranean ant-nest beetle. This species spends most of its life inside the nests of *Pheidole pallidula* (Nylander, 1849) (Hymenoptera, Formicidae), where it exploits the colony’s most valuable resources (ant larvae, pupae, and tenerals) through a suite of sophisticated chemical and structural adaptations that allow it to evade detection and integrate seamlessly into the host colony. For these reasons, *P. favieri* has recently emerged as a key model organism for studying host–parasite interactions in eusocial systems. In this study, we investigated possible correlations between the nervous system of *P. favieri* and its remarkable morphological and behavioural adaptations, shedding light on how an extreme environment such as the ant nest may have shaped the beetle’s brain. Our results, although requiring more in-depth analysis, reveal an exceptional development of the central body and the antennal lobes, which rank among the largest recorded across all insect species studied to date. We also report two previously undescribed morphological asymmetries affecting the optic lobes and mushroom bodies. Together, these findings provide new insights into the neuroanatomy of carabid beetles and, more broadly, into the biology of a unique model of ant parasitism, advancing our understanding of the evolutionary adaptations that characterise the highly specialised Paussinae subfamily, laying down the basis for further analysis.

## Simple Summary

Ant nests are among the most challenging environments for other insects to exploit. Some beetles have evolved remarkable adaptations that allow them to enter ant colonies, avoid attack, and live alongside their hosts. One of the most specialised examples is *Paussus favieri*, a rare beetle that spends almost its entire life inside ant nests, where it feeds on the ants’ young while using chemical signals and behaviour to remain accepted by the colony. Despite its extraordinary lifestyle, little is known about how its nervous system has been shaped by this extreme selective pressure. In this study, we produced the first three-dimensional description of the brain of *P. favieri* using X-ray imaging. We found that the brain regions involved in processing smells and coordinating complex behaviours are exceptionally large compared with those of other insects, while other regions are relatively reduced. We also observed unusual anatomical differences between the left and right sides of the brain that deserve further investigation. These early findings provide new insights into how living inside ant colonies may shape the evolution of the insect brain and establish a foundation for future studies on the nervous system of myrmecophilous insects.

## 1. Introduction

*Paussus favieri* Fairmaire, 1851 (Coleoptera, Carabidae, Paussinae) is a specialised myrmecophilous nest parasite belonging to the ground beetle subfamily Paussinae, which contains the highest number of myrmecophilous species among Coleoptera, including over 600 species [1]. This group is renowned not only for its evolutionary adaptations for interacting with ants but also for its remarkable explosive defensive system, which has earned them the name of “flanged bombardier beetles” [2,3]. *Paussus favieri* is one of only two European Paussinae species, and the research conducted on this rare model has significantly contributed to our understanding of their biology [4] and functional anatomy [5,6].

The adults of these beetles spend most of their lifetime inside the nests of *Pheidole pallidula* (Nylander, 1849) (Hymenoptera, Formicidae), feeding on ant larvae, pupae and tenerals while also receiving parental care from the host ant workers during their own larval stages, thanks to chemical and behavioural adaptations evolved in both ontogenetic phases [7]. Adults of *P. favieri* have modified club-shaped antennae that enclose exocrine glands producing attractive and pacificatory substances for their hosts [8]. A similar strategy is reported in *P*. *favieri* larvae [9], which possess a terminal disk resulting from a modified abdominal apex that can attract ant workers [7]. It has also been hypothesised that chemical mimicry or camouflage occurs due to the partial similarity in the cuticular hydrocarbon profiles of beetles and their host [4,7]. The ethological adaptations further contribute to the integration of this ant nest parasite into ant nests. In particular, *P. favieri* is capable of mimicking the stridulation sounds of workers or even queen host ants, thereby exploiting the ant vibrational communication channel to solicit trophallaxis or to mimic stridulation across different hierarchical levels within the colony [2,7].

Studying the morpho-functional characteristics of the brain is key to understanding what kind of stimuli are perceived and how they are processed according to their ecology, as well as providing insight about their evolution [10,11,12]. Coleoptera, with over 400,000 described species, exemplify insect evolutionary radiation and phenotypical diversity, but their brain functional organisation, directly related to specific eco-ethological adaptations is still largely unknown, with only few works exploring its structure [13,14,15,16,17,18,19,20,21,22,23,24,25,26]. The brain of ground beetles has never been quantitatively characterised: most existing studies focus on only a few brain regions, and analyse them using only classical histological techniques [21,27].

The present study takes an innovative approach to the analysis of the insect central nervous system by employing X-ray micro-computed tomography (micro-CT) as the primary method for reconstructing and examining neuroanatomical features of *P. favieri*. We aim to investigate the possible relationships between brain morphology and the parasitic lifestyle of this beetle, checking whether the extreme selective pressures that shaped the evolution of the ethological and anatomical adaptations of *P. favieri* are also reflected in the organisation of their nervous system. To this purpose, we compare the brain morphology of *P. favieri* with that of five other insect species, including two coleopterans. We also investigated whether our ultra-specialised model species exhibits a neural simplification trend similar to that observed in another highly specialised myrmecophilous beetle, *Claviger testaceus* Preyssler, 1790 (Coleoptera, Staphylinidae, Pselaphinae) [28], in which the protocerebral component is markedly reduced.

## 2. Material and Methods

Material examined: 3 specimens of *Paussus favieri*, 2 males (1M and 2M) and 1 female (1F), collected in Spain, near Tarifa in 2019, in nests of *Pheidole pallidula*.

### 2.1. Micro-CT

Micro-computed tomography (micro-CT) is a non-destructive analytical method that permits the acquisition of serial virtual thin slices revealing the internal anatomical features of biological samples. This technique employs X-rays to generate a series of two-dimensional radiographs at incremental rotation angles, which are used to provide a complete 3D reconstruction of the analysed sample. Recently, this technique has been adopted in entomological research to study the insect brain [29].

**Figure 1:**
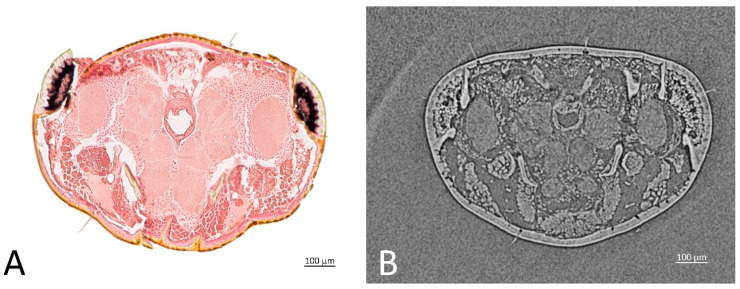
Comparison of the frontal section of two different *P. favieri* males, samples 1M and 2M, through classical histological preparation (**A**) and through micro-CT scan (**B**).

The contrast in the radiograph, arising from differences in X-ray absorption, depends on the composition and density of each biological tissue; such a setup allows the capture of multiple projection images that represent the specimen’s internal and external structure from all viewing angles. This approach avoids any damage that might occur during the preparation of classical histology slices or during dissections (Figure 1).

For tomographic analysis, 2 samples (2M and 1F) were prepared. Sample 1F was decapitated prior to fixation, preserving only the head for the analysis, while sample 2M was fixed intact. Samples were immersion-fixed in Karnovsky fixative (4% formaldehyde, 2% glutaraldehyde in cacodylate buffer 0.1 mol L^−1^, pH 7.4) for 2 weeks at 4 °C and then gradually dehydrated through serial EtOH dilutions. Dehydration began with a first step in 20% ethanol for 30 min, followed by 30% and 50% ethanol for the same duration. Once the final step with 70% EtOH was completed, samples were kept at 4 °C overnight before staining with an ethylic Lugol’s solution (1% in 70% EtOH). To increase the tissue contrast and facilitate nervous system recognition, samples were left in Lugol’s solution for 5 days before halting the staining with ethanol, after which the samples were stored at 4 °C.

The computed tomography measurements were performed at the Interdepartmental Research Centre on Nanotechnology Applied to Engineering of Sapienza University (CNIS, Sapienza University of Rome), using a Xradia 610 Versa (Zeiss, Oberkochen, Germany). Specimen 1F was scanned using 3001 projections over 360°, a source voltage of 40 kV, 3 W of power, and an exposure time of 25 s, achieving a pixel size of 0.79 µm. Specimen 2M was scanned with identical settings, except for the exposure time, which was set to 8 s, and the pixel size, which was 1.87 µm. Post-processing of the acquired volumes was performed using ImageJ software (version 1.54), applying the Enhanced Local Contrast plugin CLAHE (version 1.4.1) to enhance local contrast and using TransformJ (version 4.1.0) to adjust minor discrepancies in the alignment of the two samples. Manual segmentation of the nervous system was performed in Avizo™ (version 2022.2), analysing each slice.

### 2.2. Classical Histology

One male sample of *P. favieri* (2M) has been used for classical histology analysis with hematoxylin and eosin staining. After Bouin’s fixation (picric acid 75 mL, formalin 40% in aqueous solution 25 mL, acetic acid 5 mL) for one week, the sample was washed in 40% EtOH until the Bouin solution’s colour was no longer visible and subsequently dehydrated through a graded series of ethanol. After dehydration, the sample was treated with xylene for one hour, with the solution changed twice. For paraffin embedding, the sample was submerged in Paraplast Plus as a mounting medium at 58 °C for 2 h, with this process repeated 3 times. From the embedded sample, we obtained 8.2 µm serial sections using a Slee Cut 6062 (Slee Medical, Mainz, Germany). Slices were stained using a haematoxylin and eosin staining protocol and mounted on slides using EUKITT (Vitromed, Basel, Switzerland).

Photographs were taken with BX51 microscope (Olympus, Tokyo, Japan) equipped with OM-D E-M5 digital camera (Olympus, Tokyo, Japan), using either a 20× or 40× objective.

We analysed all photographs using ImageJ (version 1.54) to calculate the surface area of the neuropils in each slice.

## 3. Results

### 3.1. Micro-CT Results

**Figure 2:**
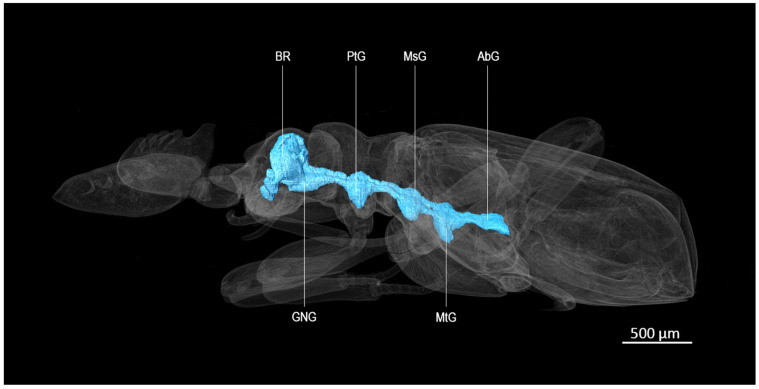
Nervous system of *P. favieri* (2M). AbG: abdominal ganglion, BR: brain, GNG: gnathal ganglia, MsG: mesothoracic ganglion, MtG: metathoracic ganglion, PtG: protothoracic ganglion.

Micro-CT scans revealed the structural organisation of the nervous system and the brain. From the reconstruction (Figure 2), it is possible to distinguish the brain and the suboesophageal ganglion from the thoracic ganglia, and a single abdominal ganglionic mass, close to the metathoracic ganglion.

**Figure 3:**
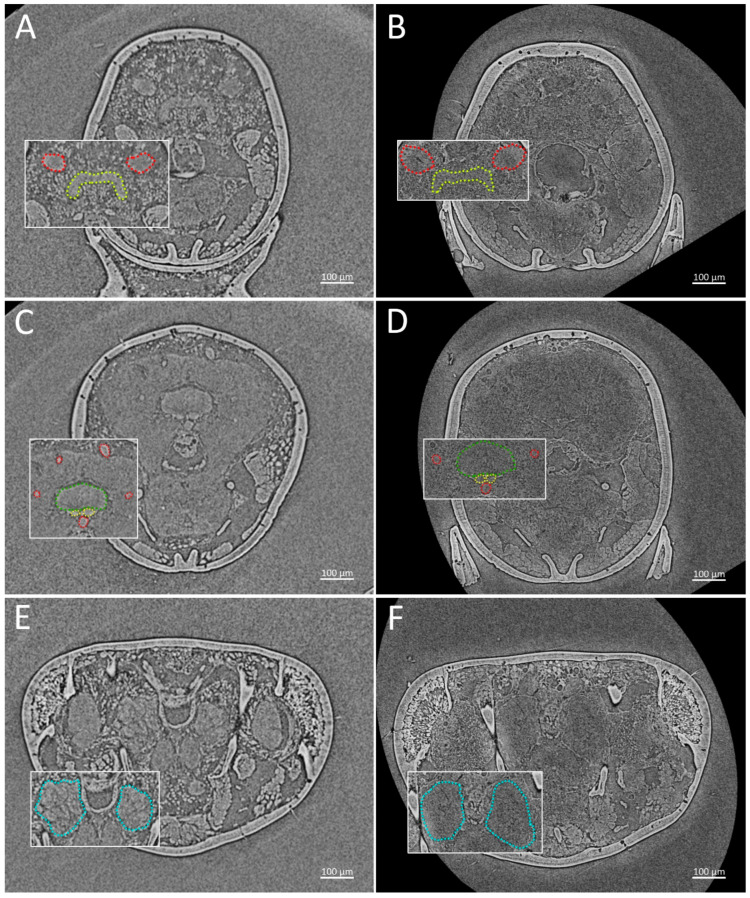
(**A**,**C**,**E**): Occipital, medial and frontal sections of the head of sample 2M. (**B**,**D**,**F**): Occipital, medial and frontal sections of sample 1F. Red: mushroom bodies; light green: protocerebral bridge; dark green: central body; yellow: noduli; light blue: antennal lobes.

**Figure 4:**
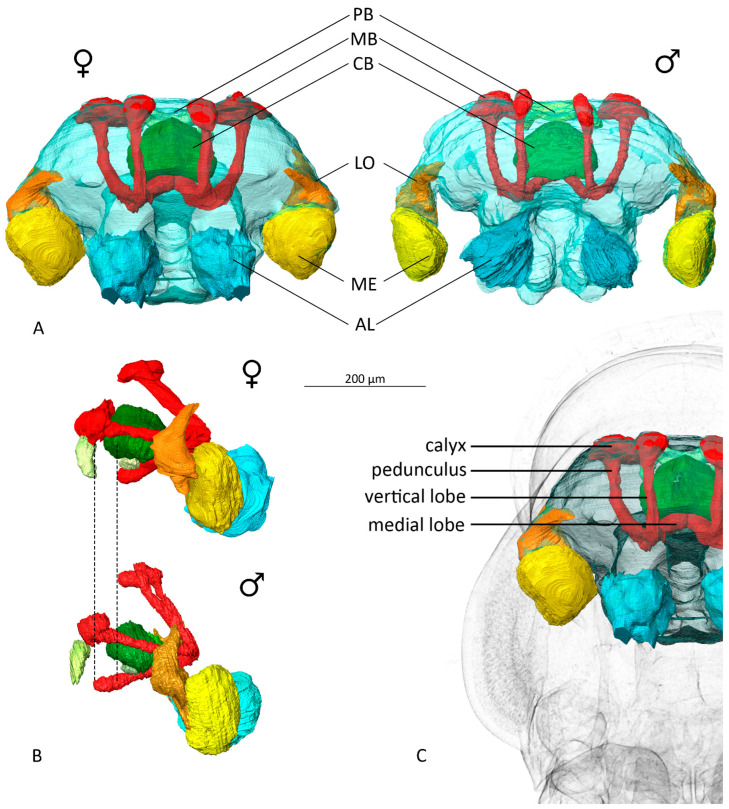
(**A**): *P. favieri*’s main brain neuropils. Antennal lobes (AL), central body (CB), lobula (LO), medulla (ME), mushroom bodies (MB), protocerebral bridge (PB). (**B**): Length difference in the left mushroom body medial lobe. (**C**): MB main sections.

The following neuropils have been reconstructed from both 2M and 1F samples (Figure 3) and quantified (Table 1): the main optic lobe components, including medulla (ME) and lobula (LO), the mushroom bodies (MB), the antennal lobes (AL) and the central complex (CX), including the central body (CB), the protocerebral bridge (PB) and the noduli (NO) (Figure 4).

The optic lobes, which consist of medulla and lobula, make up for 35.4% of the total main neuropil volume in the female and more than 41.07% in the male; lobula and accessory medulla have not been separated in the segmentation process due to the lack of resolution. The left lobula in the scanned sample shows two upward protrusions, forming a recognisable bifid shape, while the right one shows only one (Figure 4A).

Mushroom bodies’ relative volume accounts for 14% of the relative neuropil volume in the female and about 12% in the male. From both of our scans, the morphology of the vertical and medial lobes does not show any secondary protrusion. The left medial lobe is notably longer than the right lobe in both sexes, although in the male samples the left medial lobe protrudes over the central body, while in the female sample this characteristic is not present (Figure 4B), terminating right under the fan-shaped body and not extending beyond the noduli. Calyxes show a single calyx structure.

Central body is particularly developed in both sexes, representing more than 12% of the relative volume. Noduli are visible and ventrally attached to the central body.

Antennal lobes’ relative volume represents 32.5% of the main neuropil volume; in our male sample the antennal lobes do not show an equal volume, as the right lobe is 32% bigger than the left lobe. No such asymmetry is present in the female antennal lobes nor in other neuropils in both sexes.

The relative volumes are overall comparable in both the samples, although the total brain volume of the female is 49% larger than that of the male.

### 3.2. Histological Results

**Figure 5:**
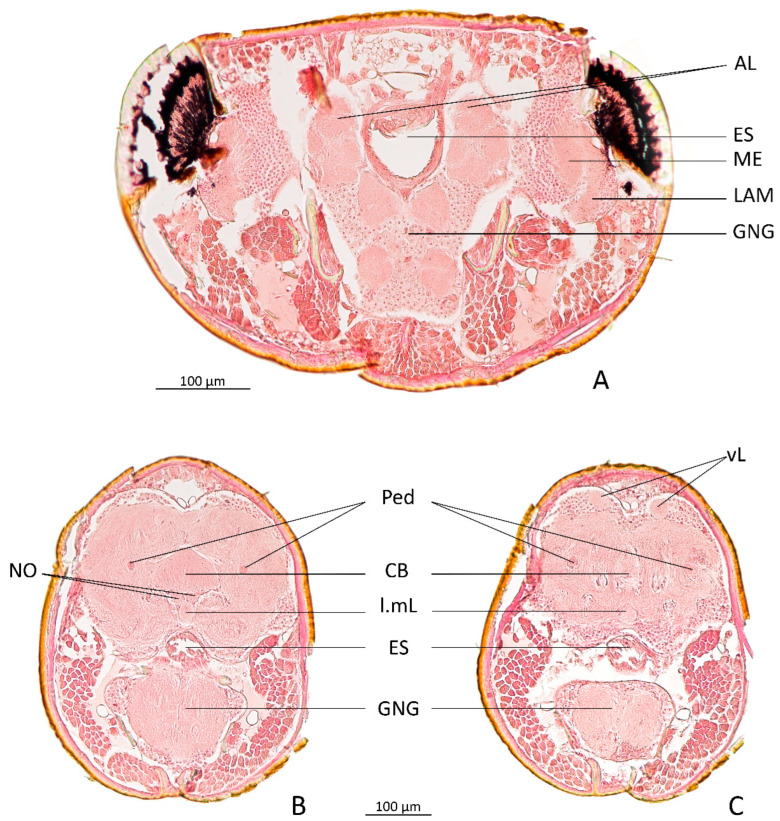
Histological sections of sample 1M. (**A**): Frontal section. (**B**): Medial section. (**C**): Occipital section. Antennal lobes (AL), oesophagus (ES), medulla (ME), lamina (LAM), peduncules (ped), central body (CB), noduli (NO), left medial lobe (l.ml), vertical lobes (vL), gnatal ganglia (GNG).

From the histological slices of sample 1M, it is possible to observe the placement and morphology of the different neuropils, with particular attention to the antennal lobe’s morphology and the mushroom body lobes (Figure 5).

Observing the occipital section of the head, the right medial lobe of the mushroom bodies extends over the central body and the noduli, indicating a similar characteristic to the micro-CT-scanned male sample.

The antennal lobes show a glomerular organisation, although we could not reconstruct the single glomerular units.

The slice thickness did not allow us to retrieve detailed morphological information, although we extracted an approximative volume by calculating the surface of each neuropil for every slice (Table 2).

## 4. Discussion

This study provides the first description of the brain morphology of a highly specialised and rare myrmecophilous beetle. Despite the limited availability of specimens and the challenging field sampling, owing to the low spatial density and restricted phenology of this species, the data obtained allowed us to explore the central nervous system of this species and to lay an important foundation for further investigations into the organisation and evolution of *P. favieri* brain. The use of micro-CT scans enabled a non-destructive three-dimensional visualisation and reconstruction of the main brain features, overcoming many of the difficulties and limitations related to classical histological procedures, which are not optimal when samples are scarce.

The ventral nerve cord shows slightly different features from other Paussinae species reported in the literature. It is well known that in coleopterans the ventral nerve cord presents different degrees of neuromere fusion [30], especially in carabid beetles, a group which can exhibit this trend in both the thoracic and ventral sections of the nervous chain [31]. In two species of the Paussinae family (*Paussus damarinus* Westwood, 1874 and *P. humboldtii* Westwood, 1874), research has previously reported a fusion starting from the third abdominal ganglion, while in our samples the fusion regards all abdominal ganglia, starting from the first one; this result is not atypical, knowing how, in ground beetles, the nerve cord length seems to be strongly related to the species body size [31]; furthermore, from our scan the abdominal ganglia seem to be close to fusing with the metathoracic ganglion.

The reconstructed neuropils were compared with the data available from previous studies and a brain atlas (Table 3 and Table 4). In particular, comparisons were made with a standardised coleopteran brain, *Tribolium castaneum* Herbst, 1797 [13], with the brain of another small beetle, *Aethina tumida* Murray, 1867 [23], and with the standard brains of *Drosophila melanogaster* Meigen, 1830, *Apis mellifera* Linnaeus, 1758 and *Schistocerca gregaria* (Forskål, 1775), calculating their relative volumes [32,33,34,35].

Comparison of the relative volumes show an increase in both antennal lobes and central complex in *P. favieri* compared with all the reference species examined. Moreover, unusual asymmetries were observed in optic lobes and mushroom bodies. Asymmetry was also observed in the male antennal lobes, with the right being 32% bigger than the left in specimen 2M and 19% bigger in specimen 1M. Further analyses on a larger number of samples should be performed to determine whether this discrepancy between the right and left antennal lobes is consistent in males.

Many features of *P. favieri* brain appear to be associated with its specialised lifestyle, such as the reduction of the optic lobes, which is often observed in edaphic species of beetles and ants [36].

In contrast, the antennal lobes show an unexpectedly large relative volume, exceeding that reported for any other species in our comparison, an enlargement likely related to the importance of chemical reception [10,36]. However, ultrastructural analyses showed that *P. favieri* antennae might have fewer chemoreceptors than those of other non-myrmecophilous beetles, as their primary role appears to be the production and dissemination of glandular secretions [5]. The large size of the antennal lobes may therefore highlight the likely importance of the olfactory processing in this species. Since the number of glomeruli has been related both to the number of detectable odours and to the accuracy with which they can be distinguished [36], further investigations are needed to clarify the relation between the possibly reduced number of chemoreceptors and the olfactory capabilities of *P. favieri*.

Furthermore, the importance of chemical perception for this beetle could be related to its close association with its host. Pheromones and colony odours play a fundamental role in the social life of ants [36,37], and *P. favieri* itself exploits the chemical trail left by the poison gland of ant queens to find its host colonies [4]. Moreover, this ant nest beetle can acquire the chemical signature of the ant queens through direct contact [4,7].

Another interesting result is the volume of the central body, which accounts for more than 12% of the total neuropil volume. Bombardier beetles possess a complex pygidial defensive apparatus that demands precise control of movement to direct the caustic substances. In the Brachininae subfamily, the defensive secretions are actively directed through pygidial movements, whereas in Paussini the insect exploits the Coanda effect by juxtaposing the flanged elytra on the opening of the gland (exit pore). It has also been observed that *P. favieri* interacts with ants by adopting acoustic mimicry strategies in addition to chemical ones [2,38]. The notably enlarged central complex volume may therefore be associated with the integration of all these abilities needed for the infiltration into the ant nest, as it is known that this neuropile is strongly associated with motor coordination and stridulation in orthoptera, as well as navigation functions in other insects [39,40,41].

Mushroom bodies are sensibly reduced, even in comparison with those of *T. castaneum.* In carabid beetles, generalist feeding species usually possess more developed mushroom bodies if compared to specialist feeding species, which are characterised by a smaller and less structurally complex calyx [14,42]. The highly specialised ecology and diet of *P. favieri*, consisting of the juveniles of *P. pallidula* colonies, may therefore be considered an adaptation related to the reduced development of this neuropile. Mushroom body morphology in *P. favieri* does not show any evidence of convergence or overlap with those of the host ant. The lack of secondary protrusion in the medial lobes is expected and consistent with the prototypical anatomy of the carabid beetle mushroom bodies [21]. Interestingly, the right medial lobe of the mushroom bodies might be associated with a dimorphic trait, being longer in both male specimens, extending over the fan-shaped component of the central body, while it is shorter in the female specimens. While this difference may represent a sexual dimorphic character, additional material will be required to confirm this interesting hypothesis. Nevertheless, to our knowledge, such an asymmetry of the medial lobes has not been previously documented in the neuroanatomical literature on insects. Although some Coleoptera species have exhibited an overlap of the right medial lobe over the left one [13,23,27], this arrangement was not observed in any of the *P. favieri* specimens analysed here, in which the two lobes are juxtaposed.

The blind cave beetle *Neaphaenops tellkampfii* Erichson, 1844 (Coleoptera, Carabidae, Trechinae) is a carabid whose entire brain has been extensively analysed using classical histological methodologies [27]. In this species, the antennal lobes and central complex are also particularly developed, similarly to what we observed in *P. favieri*. Although quantitative volumetric analyses are unavailable for *N. tellkempfii*, the aforementioned similarities suggest that a similar hypogean lifestyle and its adaptations may be reflected in a convergent brain organisation and morphology within carabids.

To date, the only available information on the brains of a myrmecophilous beetle is limited to the species *Claviger testaceus,* a highly specialised rove beetle belonging to the family that contains the greatest number of myrmecophilous species [1]. *C. testaceus* exhibits several ethological adaptations, including exploitation of the trophallaxis of its ant host (ants of the genus *Lasius*) or, alternatively, feeding on the ant larvae.

Unlike *P. favieri* and rather than actively integrating itself into the colony, *C. testaceus* strategies appear to be based primarily on the chemical mimicry of this parasite, mimicking the scent trail of dead bodies. This allows the rove beetle to be carried into the ant nest and deposited in the same compartment where the ants store the organic remains on which the larvae feed [43,44]. Although Jałoszyński et al. [45] did not specifically focus on the *C. testaceus* nervous system, they suggested that the broad development of the cephalic glandular apparatus has reduced the importance of other sensory appendages: *C. testaceus* is anophthalmic and the protocerebrum is absent. The only ganglia still detectable are the deuterocerebrum (as the antennal nerves originating from its lobes are visible) and the sub-oesophageal ganglion, which is particularly well-developed [28,45].

Although the neuropils of *C. testaceus*’s brain have not been studied either qualitatively or quantitatively, it is still possible for us to bear a comparison to the *P. favieri* brain, emphasising how different evolutionary strategies associated with extreme myrmecophily may be reflected in completely different brain architecture. Conversely to *C. testaceus*, not only does *P. favieri* retain a developed and complex brain, but some areas are hypertrophied if compared with other free-living coleoptera.

In summary, the present study revealed several unique features of the *P. favieri* brain, including asymmetries affecting the optic lobes, the mushroom bodies and potentially the antennal lobes. These preliminary results, although requiring further investigations, lay the foundations for deeper studies into the neurobiology of this rare species and other Paussinae species exhibiting similar ecologies. Furthermore, this work provides comparative data for future research into how extreme ecological specialisation can affect brain morphology and opens a new avenue for understanding the neurobiology of insect–insect parasitism.

## Figures and Tables

**Table 1:** Antennal lobes (AL), mushroom bodies (MB), central body (CB), noduli (NO), protocerebral bridge (PB), medulla (ME), lobula (LO).

*Paussus favieri* ♀			*Paussus favieri* ♂		
Neuropile	Volume $\left(\mu m^{3}\right)$	Relative Volume (%)	Neuropile	Volume $\left(\mu m^{3}\right)$	Relative Volume (%)
AL (left)	992,159.1	18.88	AL (left)	461,916.2	13.18
AL (right)	894,490.1	17.02	AL (right)	676,853.3	19.32
MB (left)	389,703.4	7.41	MB (left)	216,127.2	6.17
MB (right)	366,396.5	6.97	MB (right)	196,731.9	5.61
CB	648,556.8	12.34	CB	423,818.8	12.10
NO (left)	11,962.6	0.23	NO (left)	7690.1	0.22
NO (right)	13,325.9	0.25	NO (right)	6722.3	0.19
PB	79,270.8	1.51	PB	75,102.7	2.14
ME (left)	733,623.8	13.96	ME (left)	545,526.5	15.57
ME (right)	754,267.3	14.35	ME (right)	543,819.7	15.52
LO (left)	160,007.4	3.04	LO (left)	173,772.8	4.96
LO (right)	212,594.5	4.04	LO (right)	175,898	5.02
Total neuropil volume	5,256,358.2	100	Total neuropils volume	3,503,979.5	100
Total Brain volume	2.77 × 10^7^		volume	1.85 × 10^7^	

**Table 2:** Comparison of the male samples analysed by micro-CT segmentation (2M) and histological slices (1M). Antennal lobes (AL), mushroom bodies (MB), central body (CB), noduli (NO), protocerebral bridge (PB), medulla (ME), lobula (LO).

*Paussus favieri* ♂ (2M)			*Paussus favieri* ♂ (1M)		
Neuropile	Volume $\left(\mu m^{3}\right)$	Relative Volume (%)	Neuropile	Volume $\left(\mu m^{3}\right)$	Relative Volume (%)
AL (left)	461,916.2	13.18	AL (left)	639,589.5	14.14
AL (right)	676,853.3	19.32	AL (right)	791,892.2	17.51
MB (left)	216,127.2	6.17	MB (left)	263,263.4	5.82
MB (right)	196,731.9	5.61	MB (right)	251,387.5	5.56
CB	423,818.8	12.10	CB	472,860.5	10.45
NO (left)	7690.1	0.22	NO (left)	10,814.2	0.24
NO (right)	6722.3	0.19	NO (right)	13,124.6	0.29
PB	75,102.7	2.14	PB	60,873.7	1.35
ME (left)	545,526.5	15.57	ME (left)	645,655.5	14.28
ME (right)	543,819.7	15.52	ME (right)	597,336.8	13.21
LO (left)	173,772.8	4.96	LO (left)	124,403.1	2.75
LO (right)	175,898	5.02	LO (right)	157,183.4	3.48
Total neuropil volume	3,503,979.5	100	Total neuropils volume	4,522,892.75	100
Total brain volume	1.85 × 10^7^		Total brain volume	2.15 × 10^7^	

**Table 3:** Results from all three samples, 1M, 2M and 1F. Optic lobes (OL), antennal lobes (AL), central complex (CX: central body, protocerebral bridge and noduli) and mushroom bodies (MB).

	AVERAGE VOLUME (μm^3^)	SD (μm^3^)	REL. VOL (%)	REL. SD (%)
OL	1,608,029.6	222,785.8	36.73	3.85
AL	1,485,633.5	376,869.1	33.35	2.24
CX	930,342.9	127,579.7	13.77	1.26
MB	561,203.3	176,292.1	12.52	1.63
BRAIN	4,262,907.4	222,785.8		

**Table 4:** Relative volumes of the optic lobes (OL), antennal lobes (AL), central body (CB) and mushroom bodies (MB) in *D. melanogaster* [35], *A. mellifera* [32], *Aethina tumida* [23], *S. gregaria* [34], *T. castaneum* [13] and *P. favieri* (male and female mean).

	*A. tumida* ♀	*A. mellifera* ♀	*D. melanogaster* ♀	*S. gregaria* ♂	*T. castaneum* ♀	*T. castaneum* ♂	*P. favieri* ♀	*P. favieri* ♂
OL (%)	62.1	57.91	79.65	72.67	50.06	45.05	35.4	37.4
AL (%)	21.5	8.53	9.36	9.68	22.41	24.50	35.9	32.1
CB (%)	4.8	0.91	3.34	1.67	5.64	6.62	12.4	11.3
MB (%)	11.5	32.60	7.56	15.98	21.88	23.83	14.4	11.6

## Data Availability

The tomographic data supporting the conclusions of this article will be made available by the authors on request.

## References

[1] Parker J. (2016). Myrmecophily in Beetles (Coleoptera): Evolutionary Patterns and Biological Mechanisms. Myrmecol. News.

[2] Di Giulio A., Maurizi E., Barbero F., Sala M., Fattorini S., Balletto E., Bonelli S. (2015). The Pied Piper: A Parasitic Beetle’s Melodies Modulate Ant Behaviours. PLoS ONE.

[3] Muzzi M., Di Giulio A. (2019). The Ant Nest “Bomber”: Explosive Defensive System of the Flanged Bombardier Beetle *Paussus favieri* (Coleoptera, Carabidae). Arthropod Struct. Dev..

[4] Maurizi E., Fattorini S., Moore W., Di Giulio A. (2012). Behavior of *Paussus favieri* (Coleoptera, Carabidae, Paussini): A Myrmecophilous Beetle Associated with *Pheidole pallidula* (Hymenoptera, Formicidae). Psyche J. Entomol..

[5] Di Giulio A., Maurizi E., Rossi Stacconi M.V., Romani R. (2012). Functional Structure of Antennal Sensilla in the Myrmecophilous Beetle *Paussus favieri* (Coleoptera, Carabidae, Paussini). Micron.

[6] Di Giulio A., Fattorini S., Moore W., Robertson J., Maurizi E. (2014). Form, Function and Evolutionary Significance of Stridulatory Organs in Ant Nest Beetles (Coleoptera: Carabidae: Paussini). Eur. J. Entomol..

[7] Moore W., Scarparo G., Di Giulio A. (2022). Foe to Frenemy: Predacious Ant Nest Beetles Use Multiple Strategies to Fully Integrate into Ant Nests. Curr. Opin. Insect Sci..

[8] Di Giulio A., Rossi Stacconi M.V., Romani R. (2009). Fine Structure of the Antennal Glands of the Ant Nest Beetle *Paussus favieri* (Coleoptera, Carabidae, Paussini). Arthropod Struct. Dev..

[9] Di Giulio A., Maurizi E., Hlaváč P., Moore W. (2011). The Long-Awaited First Instar Larva of *Paussus favieri* (Coleoptera: Carabidae: Paussini). Eur. J. Entomol..

[10] Gronenberg W., Hölldobler B. (1999). Morphologic Representation of Visual and Antennal Information in the Ant Brain. J. Comp. Neurol..

[11] Bouchebti S., Arganda S. (2020). Insect Lifestyle and Evolution of Brain Morphology. Curr. Opin. Insect Sci..

[12] Adden A., Garcia Dominguez S., Kliem K., Kannan K., Yuvaraj J.K., Raif T., Boronat-Garcia A., Arganda S., Talavera G., Kelber A. (2025). The Evolution of Lepidopteran Brain Morphology. J. Comp. Physiol. A.

[13] (2010). Dreyer 3D Standard Brain of the Red Flour Beetle Tribolium Castaneum: A Tool to Study Metamorphic Development and Adult Plasticity. Front. Syst. Neurosci..

[14] Panov A.A. (2010). Structure of the Mushroom Bodies in Scarabaeoidea (Insecta: Coleoptera): 1. Basal Families and Coprophagous Scarabaeidae. Biol. Bull..

[15] Hu J.-H., Wang Z.-Y., Sun F. (2011). Anatomical Organization of Antennal-Lobe Glomeruli in Males and Females of the Scarab Beetle *Holotrichia diomphalia* (Coleoptera: Melolonthidae). Arthropod Struct. Dev..

[16] Panov A.A. (2011). Longicorn Beetles (Coleoptera: Cerambycidae) Differ Considerably in the Degree of Their Mushroom Body Development. Biol. Bull..

[17] Lin C., Strausfeld N.J. (2012). Visual Inputs to the Mushroom Body Calyces of the Whirligig Beetle *Dineutus sublineatus*: Modality Switching in an Insect. J. Comp. Neurol..

[18] Panov A.A. (2012). Mushroom Bodies in the Brain of Carrion Beetles (Coleoptera, Silphidae). Entomol. Rev..

[19] Panov A.A. (2012). Leaf Beetles (Coleoptera: Chrysomelidae): Mushroom Body Simplification in the Course of Progressive Evolution of the Family. Biol. Bull..

[20] Makarova A.A., Polilov A.A. (2013). Peculiarities of the Brain Organization and Fine Structure in Small Insects Related to Miniaturization. 1. The Smallest Coleoptera (Ptiliidae). Entomol. Rev..

[21] Panov A.A. (2013). Histological Structure of Tripartite Mushroom Bodies in Ground Beetles (Insecta, Coleoptera: Carabidae). Biol. Bull..

[22] Kollmann M., Schmidt R., Heuer C.M., Schachtner J. (2016). Variations on a Theme: Antennal Lobe Architecture across Coleoptera. PLoS ONE.

[23] Kollmann M., Rupenthal A.L., Neumann P., Huetteroth W., Schachtner J. (2016). Novel Antennal Lobe Substructures Revealed in the Small Hive Beetle *Aethina tumida*. Cell Tissue Res..

[24] Immonen E., Dacke M., Heinze S., El Jundi B. (2017). Anatomical Organization of the Brain of a Diurnal and a Nocturnal Dung Beetle. J. Comp. Neurol..

[25] El Jundi B., Baird E., Byrne M.J., Dacke M. (2019). The Brain behind Straight-Line Orientation in Dung Beetles. J. Exp. Biol..

[26] Makarova A.A., Polilov A.A. (2022). Structure of the Brain of the Smallest Coleoptera. Dokl. Biochem. Biophys..

[27] Ghaffar H., Larsen J.R., Booth G.M., Perkes R. (1984). General Morphology of the Brain of the Blind Cave Beetle, *Neaphaenops tellkampfii* Erichson (Coleoptera: Carabidae). Int. J. Insect Morphol. Embryol..

[28] Jałoszyński P., Luo X., Beutel R.G. (2020). Profound Head Modifications in *Claviger testaceus* (Pselaphinae, Staphylinidae, Coleoptera) Facilitate Integration into Communities of Ants. J. Morphol..

[29] Smith D.B., Bernhardt G., Raine N.E., Abel R.L., Sykes D., Ahmed F., Pedroso I., Gill R.J. (2016). Exploring Miniature Insect Brains Using Micro-CT Scanning Techniques. Sci. Rep..

[30] Niven J.E., Graham C.M., Burrows M. (2008). Diversity and Evolution of the Insect Ventral Nerve Cord. Annu. Rev. Entomol..

[31] Heath R.V., Evans M.E.G. (1990). The Relationship between the Ventral Nerve Cord, Body Size and Phylogeny in Ground Beetles (Coleoptera: Carabidae). Zool. J. Linn. Soc..

[32] Brandt R., Rohlfing T., Rybak J., Krofczik S., Maye A., Westerhoff M., Hege H.-C., Menzel R. (2005). Three-Dimensional Average-Shape Atlas of the Honeybee Brain and Its Applications. J. Comp. Neurol..

[33] El Jundi B., Heinze S., Lenschow C., Kurylas A., Rohlfing T., Homberg U. (2009). The Locust Standard Brain: A 3D Standard of the Central Complex as a Platform for Neural Network Analysis. Front. Syst. Neurosci..

[34] Kurylas A.E., Rohlfing T., Krofczik S., Jenett A., Homberg U. (2008). Standardized Atlas of the Brain of the Desert Locust, *Schistocerca Gregaria*. Cell Tissue Res..

[35] Rein K., Zöckler M., Mader M.T., Grübel C., Heisenberg M. (2002). The Drosophila Standard Brain. Curr. Biol..

[36] Gronenberg W. (2008). Structure and Function of Ant (Hymenoptera: Formicidae); Brains: Strength in Numbers. Myrmecol. News.

[37] Sturgis S.J., Gordon D.M. (2012). Nestmate Recognition in Ants (Hymenoptera: Formicidae): A Review. Myrmecol. News.

[38] Fattorini S., Maurizi E., Di Giulio A. (2021). Interactional Behaviors of the Parasitic Beetle *Paussus Favieri* with Its Ant Host *Pheidole pallidula*: The Mimetic Role of the Acoustical Signals. Insect Sci..

[39] Huber F. (1960). Untersuchungen über die Funktion des Zentralnervensystems und insbesondere des Gehirnes bei der Fortbewegung und der Lauterzeugung der Grillen. J. Comp. Physiol..

[40] Pfeiffer K., Homberg U. (2014). Organization and Functional Roles of the Central Complex in the Insect Brain. Annu. Rev. Entomol..

[41] Turner-Evans D.B., Jayaraman V. (2016). The Insect Central Complex. Curr. Biol..

[42] Farris S.M., Roberts N.S. (2005). Coevolution of Generalist Feeding Ecologies and Gyrencephalic Mushroom Bodies in Insects. Proc. Natl. Acad. Sci. USA.

[43] Cammaerts R. (1992). Stimuli Inducing the Regurgitation of the Workers of *Lasius Flavus* (Formicidae) upon the Myrmecophilous Beetle *Claviger testaceus* (Pselaphidae). Behav. Process..

[44] Taszakowski A., Baran A., Kaszyca N., Depa U. (2015). Genus *Claviger* in the Low Beskid Mts. Nat. J..

[45] Jałoszyński P., Luo X., Beutel R.G. (2022). Evolution of Cephalic Structures in Extreme Myrmecophiles: A Lesson from Clavigeritae (Coleoptera: Staphylinidae: Pselaphinae). Cladistics.

